# Multi-Band Sensing for Dielectric Property of Chemicals Using Metamaterial Integrated Microfluidic Sensor

**DOI:** 10.1038/s41598-018-32827-y

**Published:** 2018-10-04

**Authors:** Hong Zhou, Donglin Hu, Cheng Yang, Cong Chen, Junwang Ji, Ming Chen, Yu Chen, Ya Yang, Xiaojing Mu

**Affiliations:** 1 International R & D center of Micro-nano Systems and New Materials Technology, Key Laboratory of Optoelectronic Technology & Systems Ministry of Education, Chongqing University, Chongqing, 400044 China; 2 Beijing Institute of Nanoenergy and Nanosystems, Chinese Academy of Sciences, Beijing, 100083 China; 3 The State Key Laboratory of Mechanical Transmission, Chongqing University, Chongqing, 400044 China; 4 Department of Clinical Laboratory Medicine, Institute of Surgery Research, Daping Hospital, Third Military Medical University, Chongqing, 400042 China; 5 Institute of High Performance Computing, Agency for Science, Technology and Research, Singapore, Singapore

## Abstract

The growth of the chemical industry has brought tremendous challenges to chemical sensing technology. Chemical sensors based on metamaterials have great potential because of their label-free and non-destructive characteristics. However, metamaterials applied in chemical sensing have mainly been investigated from the measurement of sample concentration or the determination of the dielectric properties at a fixed frequency. Here we present a metamaterial integrated microfluidic (MIM) sensor for the multi-band sensing for dielectric property of chemicals, which is promising for the identification of chemicals. The MIM sensor mainly consists of multiple pair of high sensitive symmetrical double split-ring resonators (DSRRs) and meandering microfluidic channels with a capacity of only 4 μL. A dielectric model has been innovatively established and experimentally verified to accurately estimate the complex permittivity and thus realize the multi-band sensing of dielectric property of chemicals. With the increase in the number of resonators in the sensor, a dielectric spectrum like curve could be obtained for more detailed dielectric information. This work delivers a miniaturized, reusable, label-free and non-destructive metamaterial-microfluidic solution and paves a way of the multi-band sensing for dielectric property of chemicals.

## Introduction

In the last few years, liquid chemicals have been widely used in industry and laboratories. The identification and classification of such wide variety of liquid chemicals is a considerable challenge in both industry and academia, which compels the establishment of globally harmonized systems to solve it, meanwhile presenting a tremendous opportunity for the development of chemical measurement technology. In some cases, especially in the measurement of toxic, volatile, combustible chemicals, there exist risks of explosion, burning and poisoning during the measurement process^1^. Therefore, it is of great importance to develop various technologies to enhance the ability to detect chemicals and thereby reduce the risk of accidents.

So far, there have been many technologies to achieve chemical sensing, including acoustic^2,3^, optical^4–6^, pure chemical^7^ and electromagnetic^8,9^ detection. In ultrasonic sensors, the transmitter sends a discontinuous signal to the liquid, and then the echo signal is detected to obtain information including time of flight, signal amplitude, and further determines the property of sample based on the theory of Hale^10^. Although this method is rapid and precise, it can only achieve the measurement of the amount of analyte. Optical sensors utilize the sensitivity of the analyte to a particular wavelength to obtain information on wavelength drift or spectral changes, while possessing the advantage of no electrical connection^11^. However, their huge size makes it difficult to integrate into the miniaturized system.

Recently, the enormous successes of metamaterials in imaging^12^, miniaturization of the antenna^13^ and stealth^14^ have attracted great attention for sensing applications in displacement^15^, thin-film thickness^16^, strain^17,18^, inductive–capacitive detection^19^ and substance analysis such as protein^20^, biomolecules^21^ and liquid chemical^22^. Considering that microfluidic technology is widely used in the microscopic field, such as chemical reactions^23^, microanalysis^24^ and single-cell analysis^25^, one promising approach to develop a label-free and non-destructive chemical measurement technique is to combine microfluidic technology with metamaterials. This integration provides benefits for many studies, such as DNA analysis^26,27^, chemical identification^28–30^ and chemical concentration measurement^31,32^. Among them, the MIM technique for chemical detection has made some breakthroughs.

Previously published approaches to measure various chemicals using this technology include single and dual frequency measurement methods. For single frequency measurement method, it can only measure the concentration of different chemicals one by one either in a flexible paper or a hard substrate^31–35^, which limits the efficiency of this method. Moreover, since different mixtures may have the same frequency shift, measurements at a fixed frequency can only distinguish chemicals by frequency shift, which limits the reliability of the identification. For dual frequency measurement methods, it indeed promotes the efficiency of measuring concentrations and enables the measurement of chemicals at different frequencies, allowing for the discrimination of chemicals based on frequency shift at different frequencies^29,30^. However, previously published work did not establish a relationship between resonant parameters and dielectric characteristics to distinguish chemicals from their dielectric properties.

Here we propose a multi-band MIM sensor consisting of multiple pair of DSRRs and microfluidic channels with a capacity of 4 μL. The integration of metamaterial and microfluidics creates a bridge between samples and the electromagnetic resonance. A dielectric model has been innovatively established to estimate the relation between them, and the dielectric characteristics of samples at different frequencies obtained from the model are plotted in three-dimensional coordinates, which can be used as a diagnostic index for the identification of chemicals. Moreover, with the increase in the number of resonators in the sensor, the sensor can realize the multi-band sensing of dielectric property of chemicals, which enhances its potential in practical applications. In addition, compared with other chemical sensor using metamaterial absorber^29^, the MIM sensor possesses smaller sample volumes (~4 µL), more miniaturized size (24*15*0.6 mm^3^) and better linearity in ethanol sensing (−2.80%).

## Design, Principle and Analysis

### Device design

**Figure 1:**
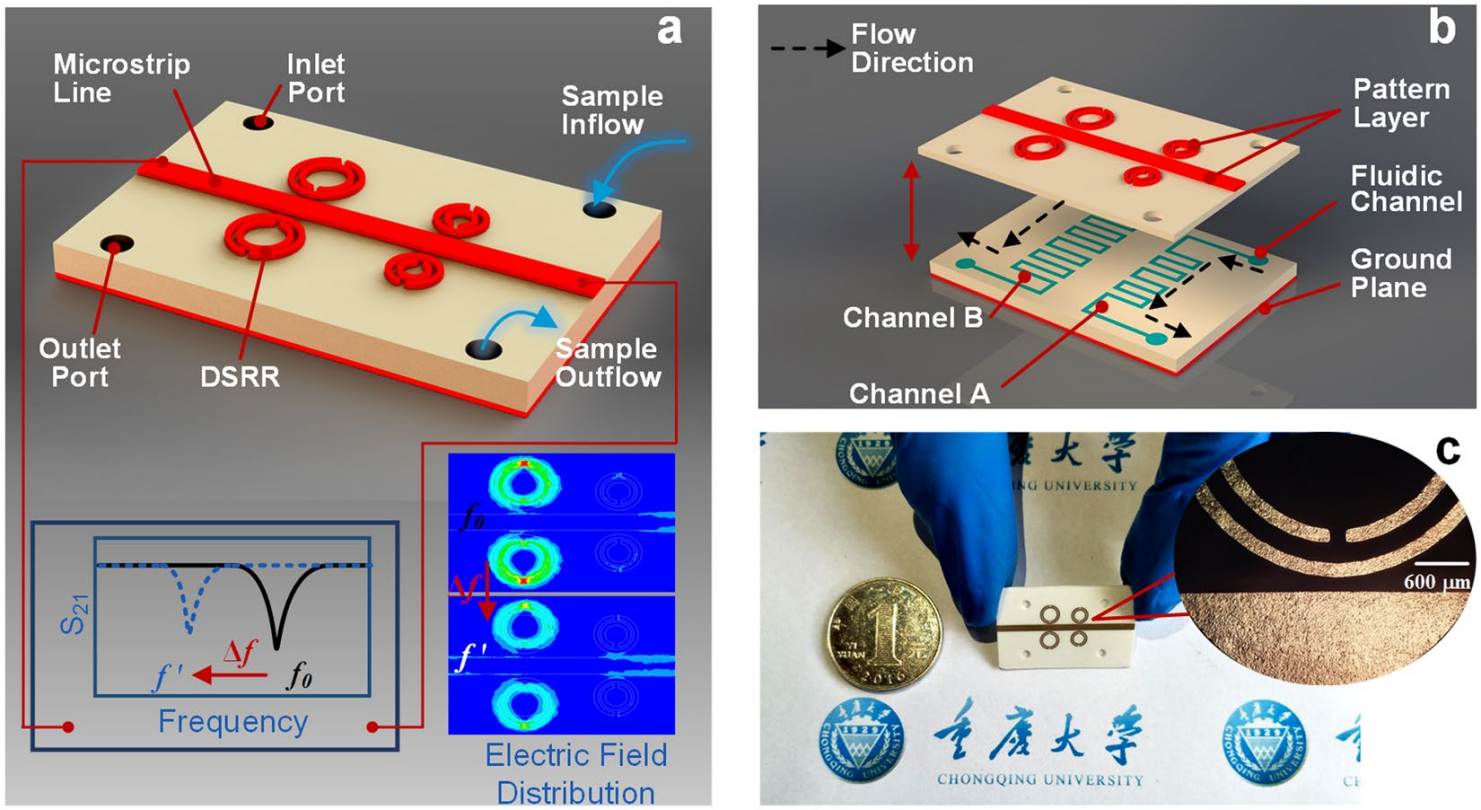
Overview of the MIM sensor. (**a**) Mock-up view: a Rogers 4003c substrate is sandwiched between a top conductive signal layer and a bottom metallic ground plane. Chemical sample in the liquid phase enters the channels from the inlet port, shifting its resonant frequency from *f*_0_ to *f* ′ due to the change of electric field distribution. (**b**) Exploded views: the microfluidic flow-channel is formed inside the substrate by adopting craft-cutting technology and an adhesive. (**c**) A photograph of our metamaterial sensor taken with a background of Chongqing University logo to show its small dimension. Inset: local enlarged optical image of the resonator near the transmission line.

The proposed sensor, illustrated in Fig. 1, consists of three layers: the signal layer including conductive DSRRs and a microstrip transmission line, the microfluidic dual-channel layer, and the fully metal-covered ground plane. The DSRRs made of two metal loops with splits on opposite side are located at close proximity to the microstrip transmission line. The purpose of the inside split ring is to generate a large capacitance in the small gap region between the rings, lowering the resonant frequency considerably and concentrating the electric field^36^. The channels in the substrate are designed in cuboid shapes and meandering paths for smooth liquid flow and larger fill area (Fig. 1(b)). The liquid sample with a max volume of only 4 μL enters from the inlet port and flow out from the outlet port after passing through the meandering channel. The bottom of substrate is fully covered with metal as ground plane with the purpose of confining the electric field induced by microstrip transmission line. When a microwave is applied to the microstrip line, a resonance occurs in the DSRRs, whose resonant characteristic is dependent on the dielectric property of substance near DSRRs. If a liquid sample is pumped into the microfluidic channel, any changes in dielectric property of sample result in redistributions of the electric field and shifts of the resonant characteristics, for example, varying resonant frequency from *f*_0_ to *f′*, as shown in Fig. 1(a).

### Principle and equivalent electrical model

**Figure 2:**
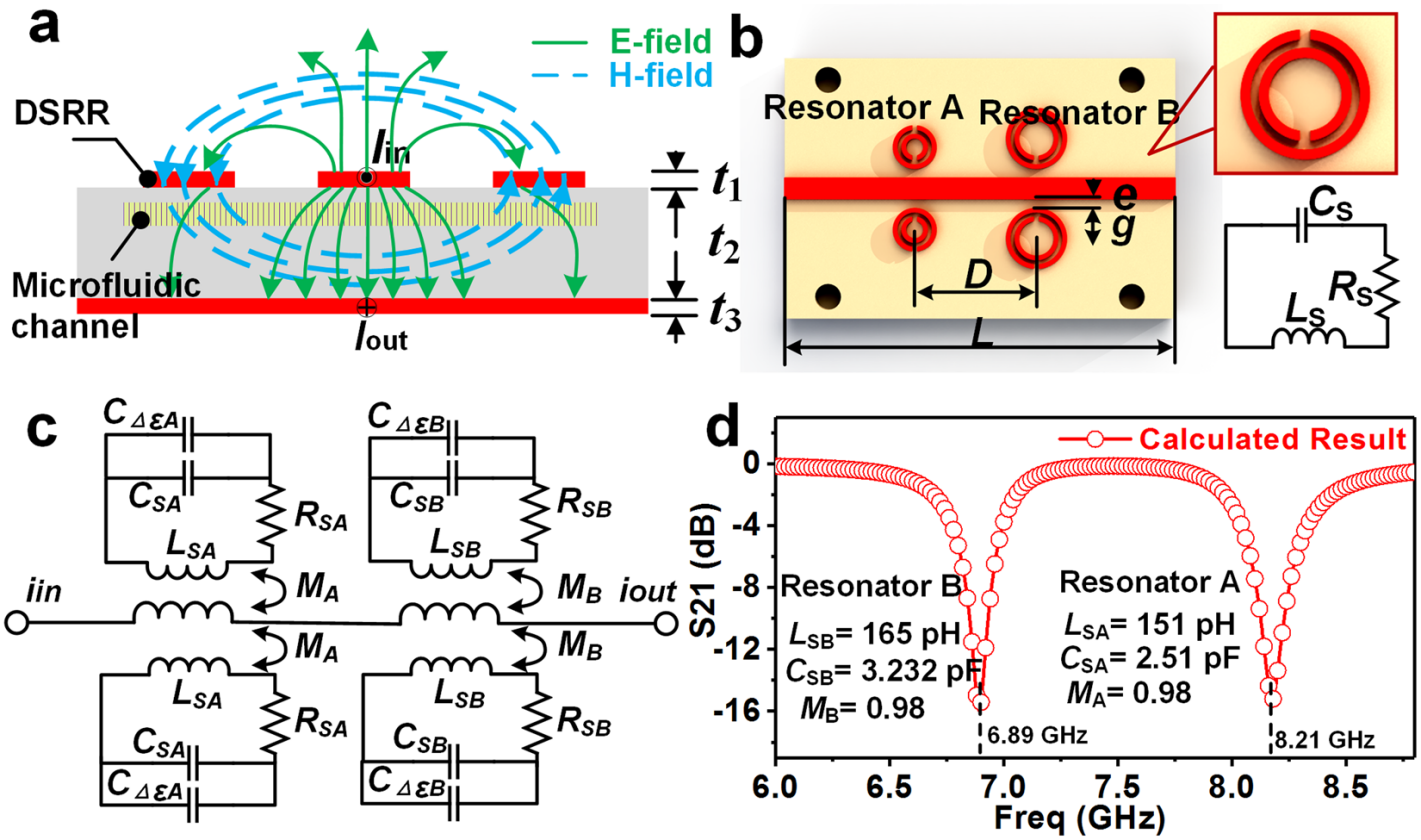
Electrical analysis of the MIM sensor. (**a**) Cross section of a microstrip transmission line with a pairs of DSRR and its electric field (E-field) and magnetic field (H-field) distribution (Thickness: t_1_ = 3.5 μm, t_2_ = 0.609 mm, t_3_ = 3.5 μm). (**b**) The equivalent circuit of DSRR and (**c**) the equivalent circuit of the microfluidic sensor. (**d**) The calculated results from the equivalent circuit in (**c**) by ADS software.

The sensing principle of the MIM sensor can be understood by investigating the electromagnetic distributions and equivalent electrical model. The electromagnetic field components occurring on DSRRs and the microstrip line are described in the Fig. 2(a). In the figure, the wave propagation in the microstrip line is a quasi-TEM mode, which is characterized by an alternating current along the strip and an alternating magnetic field around the strip. Then, an induced current is generated on DSRRs due to the alternating magnetic field component in the nearby DSRR. Remarkably, there exist two kinds of energy in the DSRR, including the magnetic energy in the ring and the energy in the split, and when they are equal, a strong resonance occurs in the DSRR. This resonance can be predicted as follows:

1 $${f}_{0}=\frac{1}{2\pi \sqrt{{L}_{s}{C}_{s}}}$$

2 $$Q=\frac{1}{{R}_{s}}\sqrt{\frac{{L}_{s}}{{C}_{s}}}$$

where *R*_*s*_, *C*_*s*_ and *L*_*s*_ represent the total resistance, capacitance, and inductance of the DSRR, respectively. From Eqs (1) and (2), it is clear that *f*_0_ and *Q* factor are dependent on *R*_*s*_, *C*_*s*_ and *L*_*s*_. Given that the geometrical dimensions remain almost unchanged after fabrication, *C*_*s*_ and *L*_*s*_ are dominated by the complex permittivity *ε* = *ε′* + *jε″* of the sample in the channel (Fig. S2 of Supplementary Information). That is, the resonant frequency and *Q* factor are a function of the sample permittivity. It can be rewritten as

3 $${\rm{\Delta }}f={F}_{freq}({\rm{\Delta }}\varepsilon ^{\prime} ,{\rm{\Delta }}\varepsilon ^{\prime\prime} )$$

4 $${\rm{\Delta }}Q={F}_{Q}({\rm{\Delta }}\varepsilon ^{\prime} ,{\rm{\Delta }}\varepsilon ^{\prime\prime} )$$

where ${\rm{\Delta }}f={f}_{ref}-{f}_{sp}$, ${\rm{\Delta }}Q={Q}_{ref}-{Q}_{sp}$, ${\rm{\Delta }}\varepsilon ^{\prime} ={\varepsilon ^{\prime} }_{ref}-{\varepsilon ^{\prime} }_{sp}$ and ${\rm{\Delta }}\varepsilon ^{\prime\prime} =\Delta {\varepsilon ^{\prime} }_{ref}-{\rm{\Delta }}{\varepsilon ^{\prime\prime} }_{sp}$, with the subscript ‘sp’ and ‘ref’ for the sample and reference, respectively. From Eqs (3) and (4), it is possible to obtain indirectly the complex permittivity of liquid samples from the measurable ∆*f* and ∆*Q*, thereby achieving chemical identification.

In quasi-static conditions, DSRR can be equivalent to an RLC resonant circuit, as shown in Fig. 2(b). Further, through quantitative analysis of the electrical properties of the DSRR (Fig. S2 of the Supplementary Information), lumped element values of the resonant circuit was derived, as listed in Table S1 of the Supplementary Information. Then, the equivalent electrical model of the MIM sensor was established by combining all electrical DSRRs units, as described in Fig. 2(c). It is worth noting the impact of changes in the dielectric properties of the sample on the resonance of the device is determined by the amount of change in capacitance (*C*_Δ*ε*_). According to the analysis of the relationship between dielectric properties and resonant frequency shifts (Fig. S3 of the Supplementary Information), the resonant frequency shift increases linearly as the permittivity Δ*ε*_*effA*_ rises. Figure 2(d) shows the calculated results of the equivalent model by circuit simulation software Advanced Design System (ADS). In the figure, there are two different resonant frequencies (6.89 GHz and 8.21 GHz) throughout the operating frequency band, which provides favorable conditions for dual-channel measurements.

### Simulation analysis

**Figure 3:**
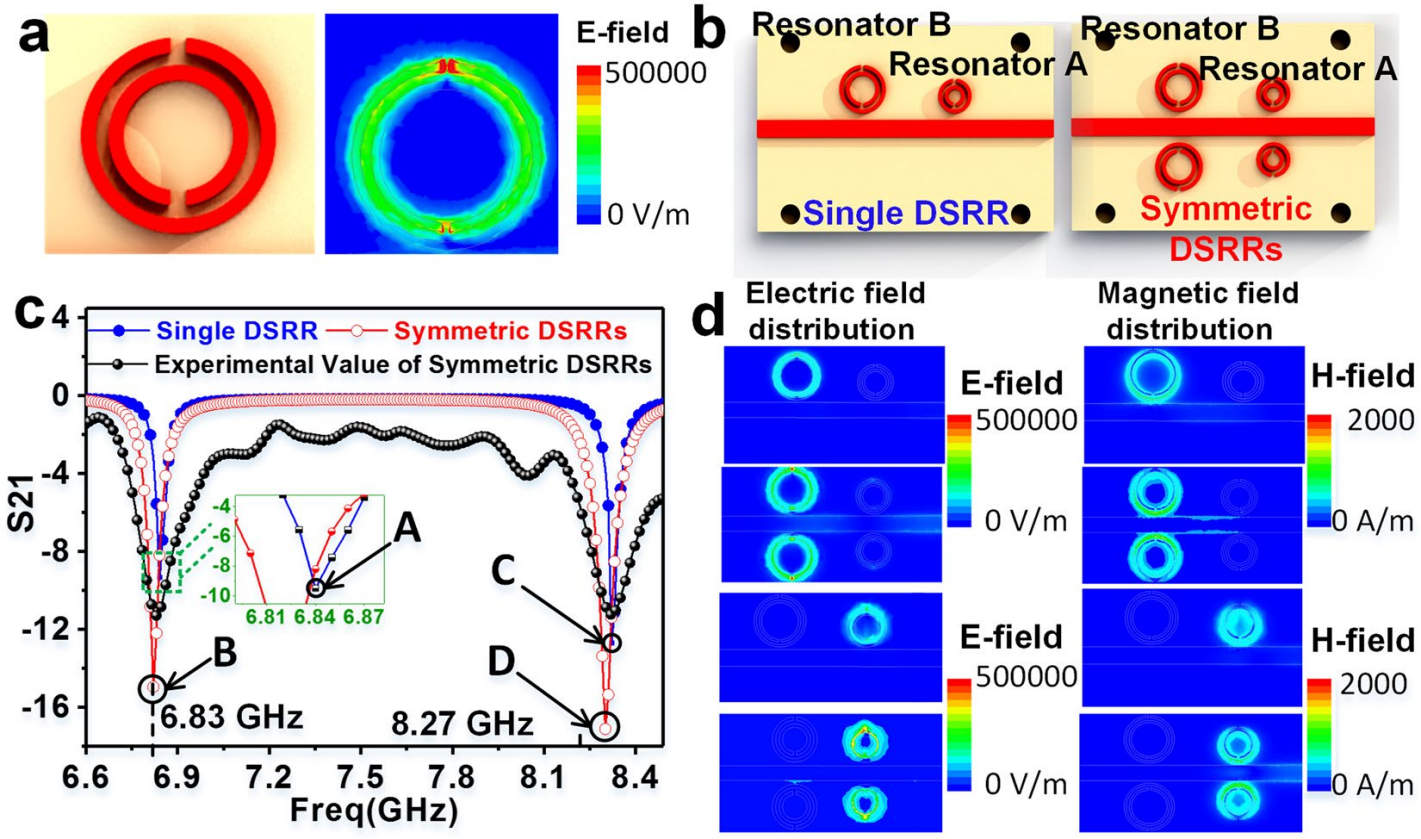
Simulation results of the sensor with various DSRRs. (**a**) The electric field distribution of DSRR. (**b**) Schematic diagram of sensors with single DSRR or symmetrical DSRRs. (**c**) The simulated and experimental S21 spectrum of single or symmetric DSRRs. Point A and B represent resonances excited by single and dual resonator B, respectively. Point C and D represent resonances excited by single and dual resonator A, respectively. (**d**) Electric and magnetic field distribution for the Point A, B, C and D in (**c**).

The performance and electromagnetic responses of the MIM sensor are simulated by the finite element simulation software ANSYS high-frequency structure simulator (HFSS). When the microwave enters into the microstrip line, the electric field is strongly coupled around DSRR, especially at the dielectric gap, as shown in Fig. 3(a). Hence, the microchannel in the substrate should be located right underneath the gap for high sensitivity. It is worth noting that the resonant frequency is inversely proportional to the radius of the resonator. Moreover, it is feasible to use the material with a lower permittivity as a substrate to enhance the sensitivity of the sensor (Fig. S4 of the Supplementary Information).

Importantly, the simulation results indicate that the proposed structure provides all the fundamental features necessary for the implementation of dual-channel chemical sensor. First, it is crucial to inspire two strong resonances in the entire frequency regime. The sensor with single DSRR and symmetrical DSRR structures, as shown in Fig. 3(b), are compared to explore the optimal structure for achieving strong resonance. The simulated transmission magnitude of the two arrangements reveals that symmetrical DSRRs structures can excite stronger resonances than single DSRR structures both on resonator A and B, as plotted in Fig. 3(c). Therefore, symmetrical DSRRs are adopted as the element of the MIM sensor instead of single DSRR. For the sensor with symmetrical DSRRs, resonant frequencies excited by resonator A and B are 8.3 GHz and 6.9 GHz, respectively, which match well with the results from the equivalent electrical model mentioned in Fig. 2(d). It is worth noting that there is a difference between the experimental and simulated S21 spectra of the sensor, which is due to fabrication errors and parasitic parameters in the measurement system, such as the parasitic capacitance between the connector and the microstrip line.

Second, it is also critical for the two resonances to remain independent of each other. When the microwave frequency in the microstrip line is approximately 6.9 GHz, the resonance occurs on the resonator B, as plotted at Point B in Fig. 3(c). It can be described as a strong distribution of the electromagnetic field around resonator B and a weak distribution of that around resonator A, as shown in the corresponding case B in Fig. 3(d). That is, the change in the dielectric constant around resonator A has little interference on the electric field distribution around resonator B, since the electromagnetic field is mainly concentrated on resonator B.

## Modeling and Discussions

Given that the dielectric properties of ethanol-water mixture have been well investigated and show a near-linear relation with volume fraction of water^37^, binary mixtures of ethanol-water with different concentrations are chosen to investigate the sensing performance of the sensor. The following study mainly consists of three parts: materials, the establishment and verification of a dielectric model, and the multi-band sensing experiment on dielectric property of chemicals.

### Materials

Ethanol (AR, >99.7%), Methanol (AR, >99.5%) were purchased from Chongqing Chuandong Chemical (Group) Co., Ltd (Chongqing, China). Acetone (AR, >99.5%), Propanol (AR, >99.7%) were purchased from Chengdu Chron Chemicals Co., Ltd (Sichuan, China). Deionized water was used to adjust the concentration of samples.

### Establishment and verification of dielectric model

**Figure 4:**
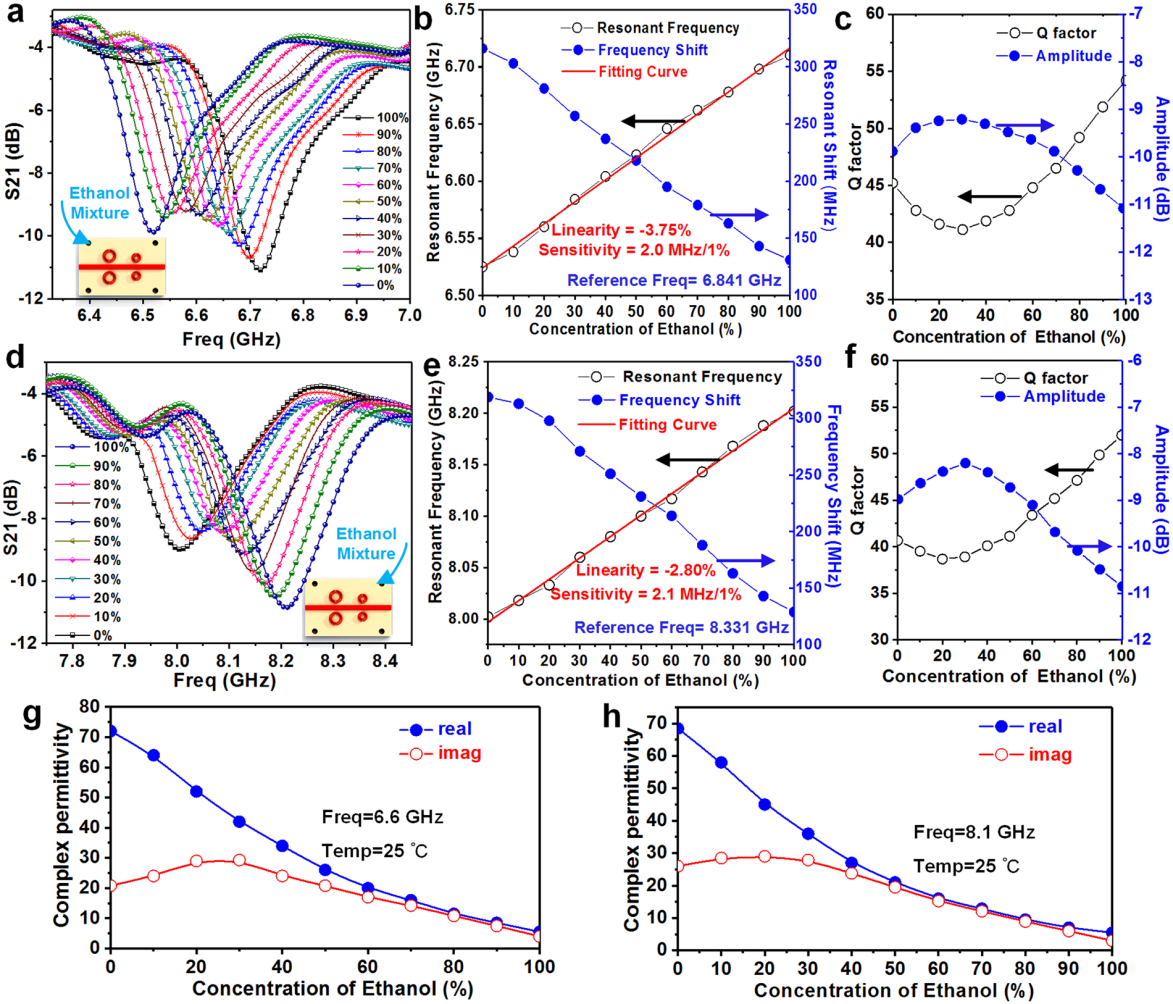
Measured results from the MIM sensor. Measured *S*_21_ for ethanol with different concentrations in channel A (**a**) and channel B (**d**), from 0% (DI water 100%) to 100%. (**b**) Corresponding resonant frequency, resonant frequency shift, (**c**) *Q* factor and resonant amplitude at different concentrations in channel A. (**e**) Corresponding resonant frequency, resonant frequency shift, (**f**) *Q* factor and resonant amplitude at different concentrations in channel B. Complex permittivity of binary ethanol-water mixtures at (**g**) 8.1 GHz (channel A) and (**h**) 6.6 GHz (channel B). The values are taken from reference 37 using linear curve-fitting of ORIGIN software. The *Q* factor is defined as *f*_0_/∆*f*_3dB_, where ∆*f*_3dB_ is the +3 dB bandwidth of the minimum value of S21.

In the following experiments, a stop-flow technique is utilized, that is, the liquid chemical is pumped into the microfluidic channel and then stopped for testing. Figure 4 shows the measured results of ethanol-water mixture with different ethanol concentration in channel A. As the concentration of ethanol in channel A increases from 0% to 100% of 10% increment per step, the resonant frequency induced by the resonator A is raised from 8.02 GHz to 8.22 GHz, as shown in Fig. 4(a). The trend of these frequency curves can be described as a “butterfly-like” shape. This shape can be understood and described by three characteristic parameters, including the resonant frequency, *Q* factor and amplitude in the frequency-S parameter curve.

First, the resonant frequencies of each curve are extracted to observe their trend, as plotted in Fig. 4(b). It indicates that the resonance frequency *f* varies linearly with ethanol concentration with the fitting curve of $y=2.1\times {10}^{-3}x+7.99$. When the sensitivity *S* of the MIM sensor is defined as the slope of the fitting curve, *S* = 2.1 × 10^6^ Hz/percentage. If the linearity *ξ* is defined as Δ*L*_max_/*y*_*FS*_, where Δ*L*_max_ is the maximum deviation between the measurement result and the fitting curve and *y*_*FS*_ represents the full scale output. Then, the linearity *ξ*_A_ of the MIM sensor is −2.80%. In order to intuitively understand the level of change in frequency, the resonant frequency is converted to frequency shift by setting the resonant frequency of empty sensor (without sample in microfluidic channel) as the reference frequency (8.331 GHz for channel A and 6.841 GHz for channel B). The max resonant frequency shift reaches 320 MHz, meaning an excellent bandwidth for sensing application. Second, the *Q* factor exhibits a non-linear change that decreases first and then begins to increase from the 20% concentration of ethanol, and the change trend of the resonant amplitude is opposite to that of *Q* factor, as shown in Fig. 4(c). These non-linear changes are attributed to the phenomenon of dielectric perturbation, which will be further analyzed in the following.

According to Eqs (3) and (4), the resonant frequency and *Q* factor are strongly related to the complex permittivity after the sensor’s fabrication. For further analysis, a model can be established to quantify the interdependency between the “butterfly-like” shaped curves and the complex permittivity of the sample. For a more accurate approximation, the model consists of a series of higher order functions, and the unknown coefficients of the function are determined by solving the equations. Take channel A as an example, the method of least squares is used to nonlinearly fit its resonant frequency and complex permittivity. Four matrices can be set up from the measured parameter (*∆f*, *∆Q*) and the reported complex permittivity (${\rm{\Delta }}\varepsilon ^{\prime}$, ${\rm{\Delta }}\varepsilon ^{\prime\prime}$) (Fig. 4g). Since the resonant shift is in a narrow band range, the effect of resonant shift on the complex permittivity is ignored. The matrices can be written as:

5 $${\boldsymbol{X}}=[\begin{array}{c}{\rm{\Delta }}{f}_{0}\\ {\rm{\Delta }}{f}_{1}\\ \cdots \\ {\rm{\Delta }}{f}_{9}\\ {\rm{\Delta }}{f}_{10}\end{array}]{,}\,\,{\boldsymbol{Y}}=[\begin{array}{c}{\rm{\Delta }}{Q}_{0}\\ {\rm{\Delta }}{Q}_{1}\\ \cdots \\ {\rm{\Delta }}{Q}_{9}\\ {\rm{\Delta }}{Q}_{10}\end{array}]{,}\,\,{\boldsymbol{Z}}^{\prime} =[\begin{array}{c}{\rm{\Delta }}{\varepsilon ^{\prime} }_{0}\\ {\rm{\Delta }}{\varepsilon ^{\prime} }_{1}\\ \cdots \\ {\rm{\Delta }}{\varepsilon ^{\prime} }_{9}\\ {\rm{\Delta }}\varepsilon {\text{'}}_{10}\end{array}]{,}\,\,{\boldsymbol{Z}}^{\prime\prime} =[\begin{array}{c}{\rm{\Delta }}{\varepsilon ^{\prime\prime} }_{0}\\ {\rm{\Delta }}{\varepsilon ^{\prime\prime} }_{1}\\ \cdots \\ {\rm{\Delta }}{\varepsilon ^{\prime\prime} }_{9}\\ {\rm{\Delta }}{\varepsilon ^{\prime\prime} }_{10}\end{array}]$$

**Figure 5:**
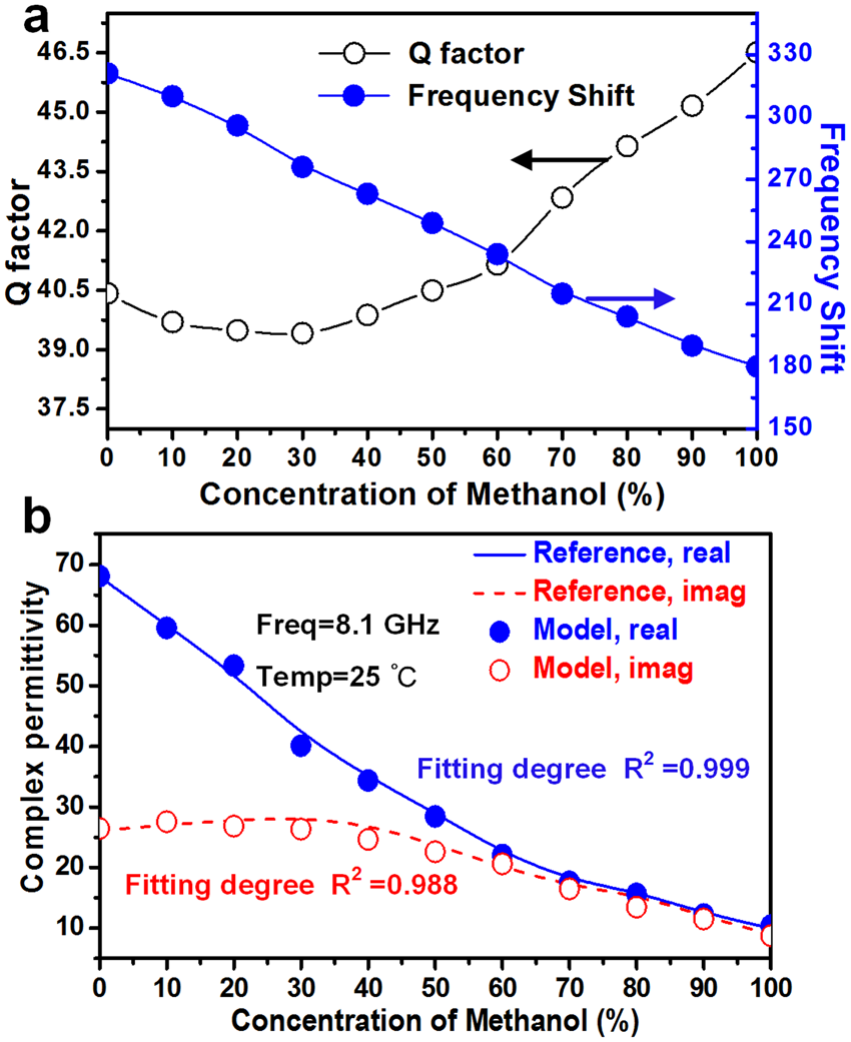
Verification of the dielectric model. (**a**) Corresponding measured resonant frequency shift and *Q* factor at different methanol concentrations in channel A. (**b**) Comparison of complex permittivity at 8.1 GHz from model estimation and literature^37^.

The fitting function can be set as

6 $${\boldsymbol{Z}}^{\prime} ={a}_{11}X+{a}_{12}{X}^{2}+{a}_{13}{X}^{3}+{b}_{11}Y+{b}_{12}{Y}^{2}+{b}_{13}{Y}^{3}$$

7 $${\boldsymbol{Z}}^{\prime\prime} ={a}_{21}X+{a}_{22}{X}^{2}+{a}_{23}{X}^{3}+{b}_{21}Y+{b}_{22}{Y}^{2}+{b}_{23}{Y}^{3}$$

where these unknown coefficients can be determined from (5) using MATLAB software. After the operation, the complex permittivity of the sample can be written as

8 $${\boldsymbol{Z}}^{\prime} =-\,{\rm{5.2943}}X+0{\rm{.0232}}{X}^{2}-{\rm{3.0205}}{X}^{3}+{\rm{0.8395}}Y-{\rm{0.1439}}{Y}^{2}-{\rm{0.0065}}{Y}^{3}$$

9 $${\boldsymbol{Z}}^{\prime\prime} =-\,{\rm{2.1203}}X+0{\rm{.0087}}{X}^{2}-{\rm{1.1348}}{X}^{3}+{\rm{68.0887}}Y-{\rm{1.6665}}{Y}^{2}-{\rm{0.0007}}{Y}^{3}$$

where the square of the correlation coefficient (*R*^2^) is 0.9944 and 0.9972, respectively, indicating that these coefficients solved by MATLAB are reasonable. Although empirical, the dielectric model builds a bridge between resonant properties and complex permittivity, thus allowing the determination of permittivity of the sample from the measurable resonance. The same research method is utilized to investigate the measurement of the sample in channel B. Figures 4(d) and 5(e,f)shows the measured results of ethanol-water mixture with different ethanol concentrations in channel B.

Similar to the previous process, an analogous dielectric model for channel B can be established by the previous algorithm, and the unknown coefficients are summarized in Table 1. All the squares of the correlation coefficient are greater than 99.2%, which reflects the reasonableness of these nonlinear fitting. It is worth noting that the linearity *ξ*_B_ = −3.75% and sensitivity *S*_B_ = 2.0 × 10^6^ Hz/percentage, which can be enhanced by more sophisticated and expensive fabrication process.

**Table 1:** Summary of the coefficients of the dielectric model.

Permittivity matrix	*a*_x1_^a^	*a*_x2_	*a*_x3_	*b*_x1_	*b*_x2_	*b*_x3_	*R* ^2^
**Z**′_A_	−5.06195	0.02225	−0.0000289	1.17620	0.47318	−0.007048	0.9944
**Z**_A_″	−1.24300	0.00472	−0.0000057	11.15725	−0.26550	−0.001672	0.9971
**Z**′_B_	−0.02400	0.000453	0.0000011	−0.49010	14.93790	0.020000	0.9995
**Z**_B_″	−4.48272	0.02303	−0.0000364	5.87300	0.12846	−0.002624	0.9922

^a^Subscript x can be substituted by 1, 2, 3 and 4.

In order to verify the dielectric model, another mixture is selected as the sample to be tested. Figure 5(a) shows the measured results of methanol-water mixtures with different concentrations in channel A. Eqs (8) and (9) are then used to calculate the complex permittivity of the mixtures under test. The calculated values from the dielectric model are plotted against reference values from literature, as shown in Fig. 5(b). The correlation coefficients between the calculated values of *ε*′ and *ε*″ and the literature values are 0.999 and 0.988, respectively, which indicates the reasonableness of the dielectric model. Small differences may be caused by the uncertainties of the measurement and can be decreased by repeated measurements and more accurate models.

### The multi-band sensing experiment on dielectric property of chemicals

Through the established dielectric model, the sensor can be used to determine the dielectric property of various chemicals at different frequencies. Specifically, samples were injected into different channels for electromagnetic detection at different frequencies. The detailed operation is described as follows (Fig. S7 of Supplementary Information).

**Figure 6:**
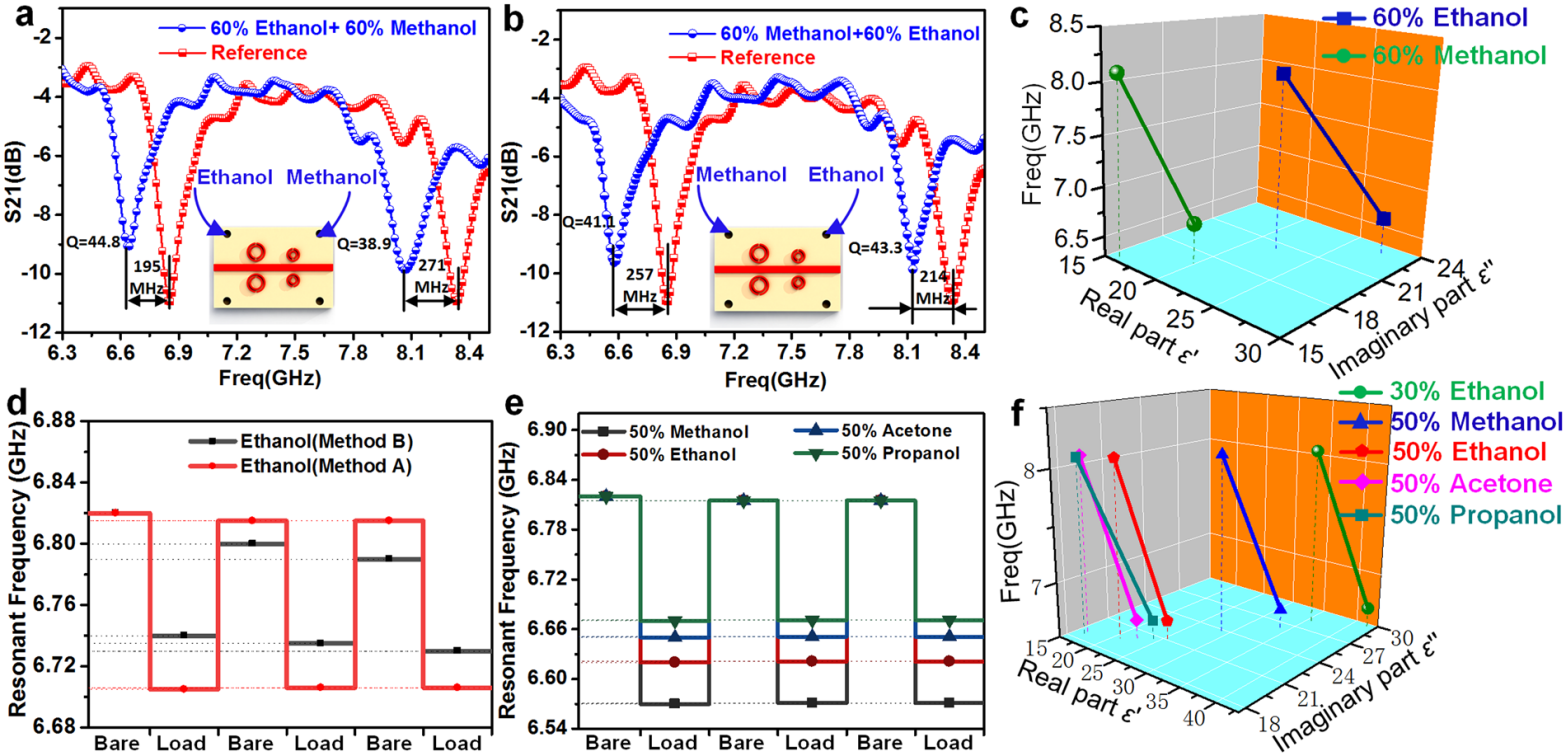
The dual-band sensing of dielectric property of chemicals using the MIM sensor. (**a**) Measured results when channel A and channel B are filled with ethanol and methanol of 60% concentration, respectively. (**b**) Corresponding results when samples are exchanged. (**c**) The dielectric characteristic line of samples, when the frequency, the real and imaginary parts of the complex permittivity are taken as the Z, X and Y axes of the coordinate system, respectively. The reproducibility of signals (**d**) when using different cleaning methods in channel A (**e**) or filling different samples in the channel A. (**f**) The dielectric characteristic line of different types of samples.

First, a 60% ethanol-water mixture (sample A) and a 60% methanol-water mixture (sample B) were pumped into channel A and B, respectively, and then the response of resonant frequency and *Q* factor could be acquired, as described in Fig. 6(a). The reference frequency was set to the frequency when the microfluidic channel is empty. By substituting the values of resonant frequency and *Q* factor into the dielectric model, the complex permittivity of the sample A at 8.1 GHz (Channel A) is approximately *ε*′ = 16.1 and *ε*″ = 15.2, and that of the sample B at 6.7 GHz (Channel B) is *ε*′ = 27.9 and *ε*″ = 22.9. Next, the sample A and B were exchanged and pumped into channel B and channel A, respectively, and then the response of resonant frequency and *Q* factor could be acquired, as shown in Fig. 6(b). According to the model, the permittivity of the sample B at 8.1 GHz (Channel A) is approximately *ε*′ = 22.5 and *ε*″ = 20.1, and that of the sample A at 6.7 GHz (Channel B) is *ε*′ = 19.9 and *ε*″ = 17. All the results are listed in Table 2.

**Table 2:** Summary of the permittivity of the samples under test.

Cases	*ε*′ (6.7 GHz)	*ε*″ (6.7 GHz)	*ε*′ (8.1 GHz)	*ε*″ (8.1 GHz)
Sample A	19.9	17	16.1	15.2
Sample B	27.9	22.9	22.5	20.1

These data indicate that the dielectric properties of the sample at various frequencies are different, especially when recorded in 3 axes of the coordinate system, each sample has its own dielectric characteristic line, as plotted in Fig. 6(c), which clearly distinguishes the samples. That is because each material has a unique complex dielectric spectrum in the radio/microwave frequency range^37^. It means that the permittivity is a variable diagnostic index for the distinguishment of chemicals. To verify this finding, 5 more samples obtained their own dielectric characteristics through the above method, as shown in Fig. 6(f). It is clear that the dielectric characteristics of each chemical are different, indicating that this technology has the potential to identify various chemicals.

The reproducibility of signals is critical when changing the filling chemicals in fluidic channels. Considering the relatively small volume of the sample, there are two ways of cleaning. One is drying after thorough washing with deionized water (Method A), and the other one is simple drying without washing (Method B). Figure 6(d,e) show the reproducibility of signals when using the two cleaning methods to detect ethanol in channel A. Obviously, the rinsing and drying combined cleaning method allows the measurement to achieve better signal reproducibility. This is due to the fact that the sample in the fluidic channel is soluble and can be carried away by deionized water. When using Method A to clean other samples in the channel, the test still has good signal reproducibility (within 5 percent deviation), as described in Fig. 6(e).

**Figure 7:**
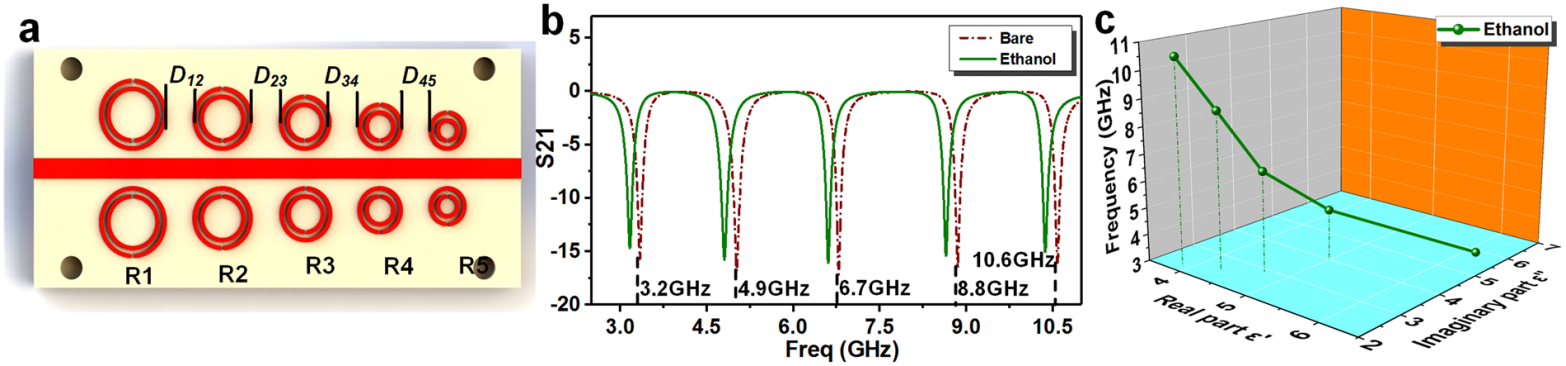
The multi-band sensing of dielectric property of chemicals using the MIM sensor with multiple resonators. (**a**) The schematic diagram of MIM sensor with multiple resonators. (**b**) The corresponding simulated frequency response of the MIM sensor, when the channels are bare or filled with ethanol. (**c**) The dielectric spectrum of ethanol obtained from the MIM sensor.

In order to improve the reliability and practicality of the measurement, more resonators of different sizes can be cascaded to obtain more information about permittivity at different resonant frequencies, as shown in Fig. 7. Figure 7(a) shows the schematic diagram of MIM sensor with multiple resonators, and the dimension of each resonator is listed in Table S3 of the Supplementary Information. Remarkably, since the electromagnetic energy of the resonator is almost distributed at the split of the resonator, the interference induced by the adjacencies between the resonators is small (Fig. S8 of Supplementary Information), which means that the resonators can work independently regardless of the distance between them. Meanwhile, in order to reduce the size of the sensor and avoid electrical contact between the resonators due to manufacturing errors, the minimum spacing *D* between the two resonators can be set to 3 mm. Compared with the dual-band sensor, this multi-band sensor integrates more resonators but has the same working principle, so it is efficient to study its performance in chemical sensing through simulation analysis. Figure 7(b) shows the corresponding frequency response of the multi-band sensor, when the channels are bare or filled with ethanol. Then, the dielectric characteristic line of ethanol at multiple frequencies can be obtained from the established dielectric model, as shown in Fig. 7(c). Obviously, as the number of points in the coordinate system increases, the dielectric line will be approximately a dielectric spectrum, which is very important in chemical identification and many engineering applications. Compared with commercial coaxial probe technology, this method of measuring the dielectric properties of liquids has the advantages of low cost, non-contact and less usage of sample.

## Conclusions

In this paper, we have demonstrated a MIM sensor with the capability of multi-band sensing for the dielectric property of various chemicals. The main component of the sensor is symmetrical DSRRs structure, which enhances the resonant intensity and sensing capability of the resonator (320 MHz shift) compared to single DSRR. By means of nonlinear curve fit and theoretical derivation of resonance, a dielectric model has been established and experimentally verified to obtain complex permittivity from the measured “butterfly-like” shaped result curves. The dielectric characteristic line of some samples, including but not limited to ethanol, methanol, acetone, and propanol, have been determined by the model and showed significant differences, which can serve as a diagnostic indicator for distinguishing chemicals. Furthermore, the sensor is expanded to integrate multiple resonators to acquire the dielectric spectrum of ethanol, which enhances its potential in chemical analysis and practical applications. This work delivers a miniaturized, reusable, label-free and non-destructive metamaterial-microfluidic solution and paves a way of the multi-band sensing for dielectric property of chemicals, which potentially promotes the development of analytical chemistry.

## Method

### Simulation

Frequency domain simulations of the MIM sensor were performed with the finite element simulation software ANSYS high-frequency structure simulator (HFSS). The structure of the sensor was placed in a vacuum box in the HFSS. The six sides of the box were assigned radiation boundary conditions. The type of excitation was wave port.

### Fabrication

The sensor was fabricated by utilizing standard chemical wet etching and photolithography on a conventional printed circuit board. The ground plane, DSRRs and microstrip line are made from copper coated with 3.5 µm thick Ni/Au layer. Rogers 4003c with thickness of 0.203 mm was chosen as the substrate because it has an ultra-low dielectric loss (*ε*_r_ = 0.0027) than some other materials such as FR4 (*ε*_r_ = 0.02). The microfluidic flow-channel was formed by adopting craft-cutting technology and positioned to pass right underneath the gaps of DSRR. Finally, the two layers were attached to each other by an epoxy adhesive layer. As epoxy adhesive can coexist with some organic solvents after curing, the sensor has a wide range of detection and can be applied to sense methanol, ethanol, acetone and so on. The detailed fabrication sequences are described in the Fig. S6 of Supplementary Information.

### Experimental setup

**Figure 8:**
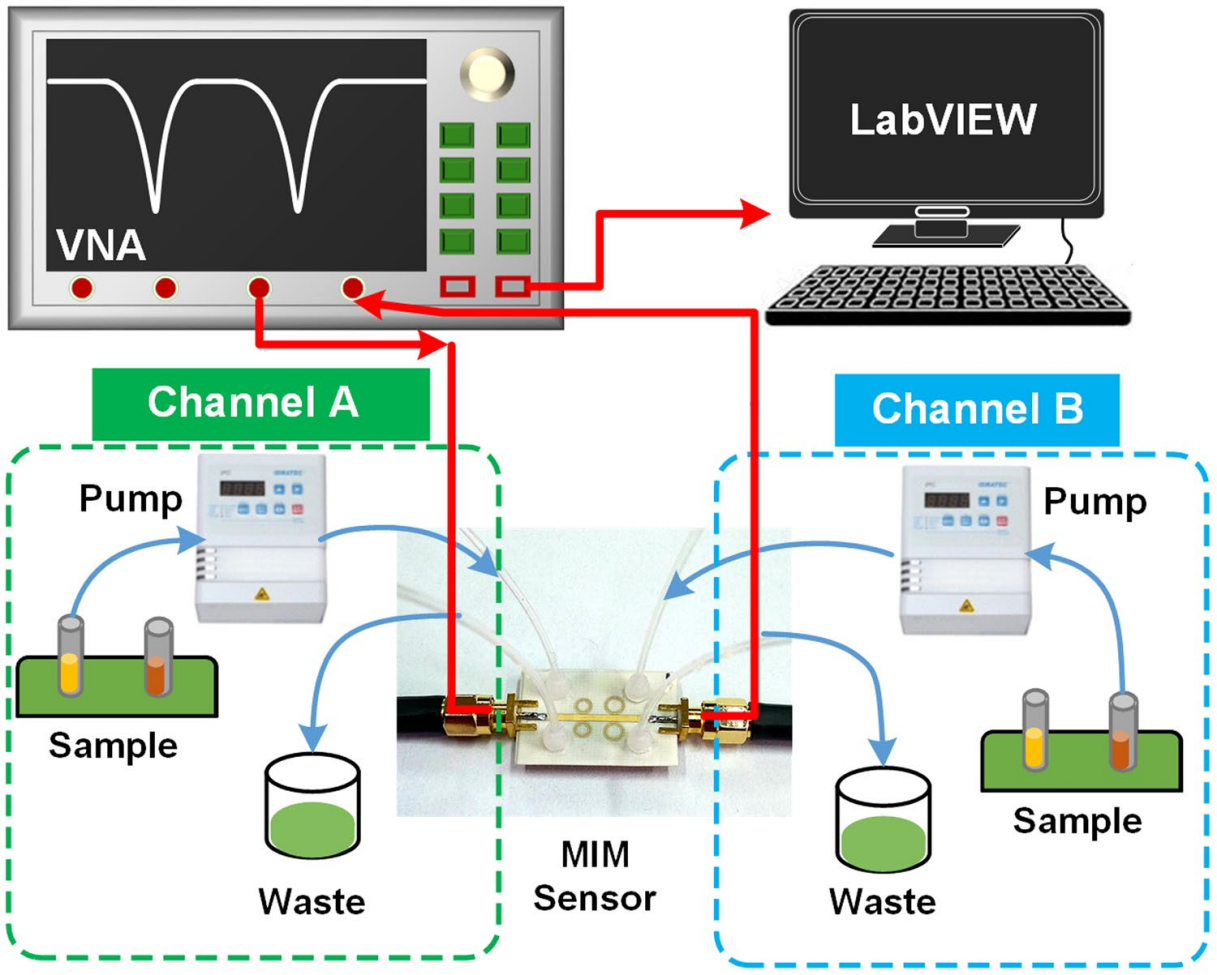
Diagram of the experimental setup. The blue solid line represents the flow path of liquid chemicals, and the red solid line indicates signal transmission path. Channel A and B correspond to resonator A and B respectively. All measurements are carried out at room temperature (25 °C).

In order to investigate the performance of the MIM sensor, an experimental setup was built as shown in Fig. 8. The setup mainly consists of a peristaltic pump, the sensor, a Keysight E5080A ENA vector network analyzer (VNA) and a computer with LabVIEW installed. Liquid chemicals were filled with microfluidic channel under pump-powered conditions. Any change in the permittivity of the liquid sample will cause the occurrence of resonant frequency shift. The resonant frequency drift of the sensor was monitored by VNA, which can perform frequency swept measurements in the desired frequency range and obtain the scattering parameters (*S* parameters). Meanwhile, the VNA was connected to the computer to obtain the information of the resonant frequency in the time domain, which helps to study the dynamic characteristics of the sensor, including response time and repeatability.

## Electronic supplementary material

Supplementary Information
