# Supplementary material for: Multi-Band Sensing for Dielectric Property of Chemicals Using Metamaterial Integrated Microfluidic Sensor
Source: Sci Rep. 2018 Oct 4;8:14801. doi: 10.1038/s41598-018-32827-y (PMC6172240; doi:10.1038/s41598-018-32827-y)
Supplement: Supplementary file 1 — Supplementary Information [file 41598_2018_32827_MOESM1_ESM.pdf]

Supplementary Information

Multi-Band Sensing for Dielectric Property of Chemicals Using Metamaterial Integrated Microfluidic Sensor

Hong Zhou[a,c], Donglin Hu[a,c], Cheng Yang[d], Cong Chen[a,c], Junwang Ji[a,c], Ming Chen[d,*], Yu Chen[e], Ya Yang[b,*], Xiaojing Mu[a,c,*]

[a] International R & D center of Micro-nano Systems and New Materials Technology, Key Laboratory of Optoelectronic Technology & Systems Ministry of Education, Chongqing University, Chongqing 400044, China

[b] Beijing Institute of Nanoenergy and Nanosystems, Chinese Academy of Sciences, Beijing 100083, China

[c] The State Key Laboratory of Mechanical Transmission, Chongqing University, Chongqing 400044, China

[d] Department of Clinical Laboratory Medicine, Institute of Surgery Research, Daping Hospital, Third Military Medical University, Chongqing 400042, China

[e] Institute of High Performance Computing, Agency for Science, Technology and Research, Singapore

Corresponding Authors:

* Xiaojing Mu, Tel: 86-15902396712, Email: mxjacj@cqu.edu.cn;

* Ya Yang, Email: yayang@binn.cas.cn;

* Ming Chen, Email: chming1971@126.com;

1. Various designs for microfluidic channels

In order to investigate the effect of the microfluidic channel on the efficiency and sensitivity of the sensor, three shapes of microfluidic channels are proposed and simulated by finite element simulation software, as shown in Figure S1a~c. It is worth noting that the resonators of the sensors in Figure S1a~c are identical, and the S21 spectrums are obtained by simulation software when the channels are empty or filled with ethanol (ε_r= 25), as shown in Figure S1d~f. Obvious, the sensor with a large cubic channel is more sensitive than others, which is due to the fact that more samples in the cubic channel result in larger changes in dielectric constant. However, considering that the minimum pressure required to induce flow is inversely proportional to the cross-sectional dimensions of the microfluidic channel according to the Young-Laplace equation[1], the microfluidic channel of this design suffers from a problem of uneven filling, which makes filling and cleaning of samples in the channel difficult. Meantime, although the sensor with a straight channel is more efficient and requires less volume of samples, it has a relative low sensitivity. Therefore, a sensor with meandering channel is used as the final design to ensure the smooth flow of liquids and achieve higher sensitivity of the sensor.

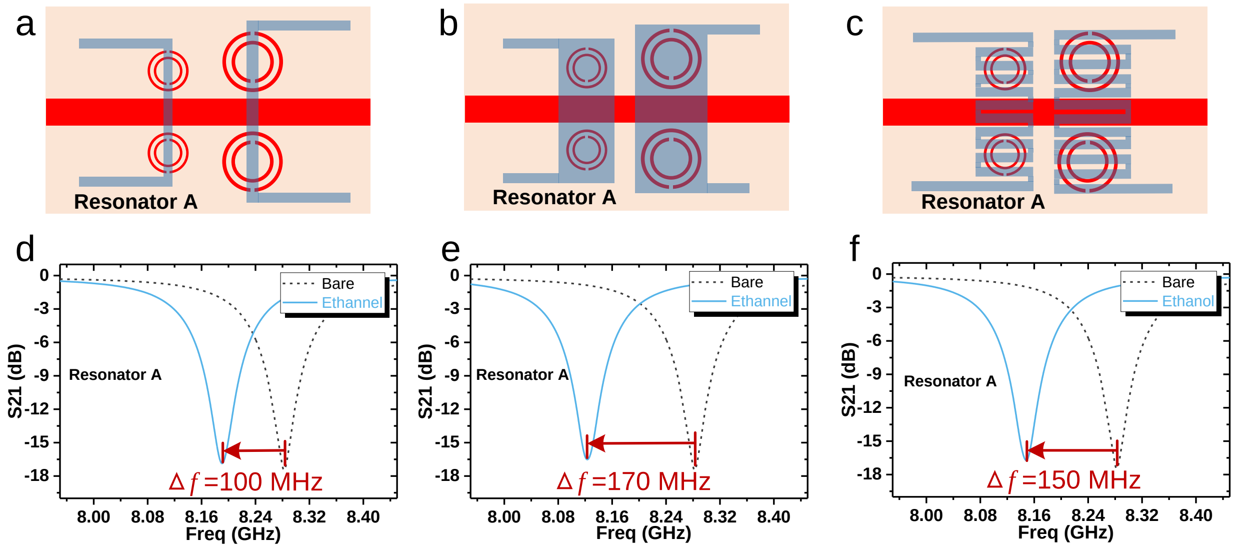

Figure S1. Several designs for microfluidic channels. (a,d) Schematic and corresponding performance of a sensor with a straight channel. (b, e) Schematic and corresponding performance of a sensor with a large cubic channel. (c, f) Schematic and corresponding performance of a sensor with meandering channel.

2. Analysis of DSRR's resistance, inductance and capacitance

As shown in Figure S2, two pairs of resonators are symmetrically located on both sides of the microstrip line, exciting two different resonances in the entire frequency regime due to their different sizes, which creates favorable conditions for the implementation of dual-channel detection. According to the electrical characteristics of the series circuit, the impedance of the resonator can be expressed as:

$$Z_s = R_s + j\omega L_s + \frac{1}{j\omega C_s} \tag{S1}$$

where $R_s = R_x R_y \big/ \left(R_x + R_y\right)$, R_x and R_y are parameters related to the radiation and Joule's losses in both loops, the DSRR element can be assumed to be perfectly conducting at this stage. L_s is determined by the inductance of the inner ring (L_y), the inductance of the outer ring (L_x) and the mutual inductance between the two rings (M). When the round loop (radius r) is made of a conductive strip (width c) and $c \ll r$, the inductance of the round loop is approximately the same as that of a round loop made of the wire (radius $r_0 = c/4$):

$$L = \mu_0 \mu r \left[\log\left(\frac{8r}{r_0} \right) - 1 \right] \tag{S2}$$

The inductance of the two rings of the resonator can be obtained using Eq. (S2) by substituting $r = k_1, k_2$, that is

$$L_{x,y} = \mu_0 \mu r \left[\log\left(\frac{8k_{1,2}}{r_0} \right) - 1 \right] \tag{S3}$$

The mutual inductance between the two rings can be approximated as:

$$L_M = \mu_0 \mu k_2 \left[\left(1-\xi\right) \log\left(\frac{4}{\xi} \right) - 2 + \xi \right] \tag{S4}$$

where $\xi = (k_2 - k_1)/2k_2$ is a small parameter (ξ < 0.25). The terms of order ξ^2 and higher have been neglected in Eq. (S4). By substituting the corresponding parameter values into Eqs. (S3) and (S4), the inductance of resonator A and resonator B can be obtained as L_{SA}= 151 pH, L_{SB}= 165pH.

The capacitance of DSRR can be divided into two parts (C_1, C_2) for analysis,

$$C_1 = C_{sx} + \frac{C_m}{2}, \quad C_2 = C_{sy} + \frac{C_m}{2} \tag{S5}$$

In Eq. (S5), the mutual capacitance of the broken loops in the DSRR can be approximated as [2]

$$C_m = \frac{(k_1 + k_2)}{2\pi} \varepsilon_0 \varepsilon \cosh^{-1}\left(\frac{2c}{d}\right) \tag{S6}$$

When the length of the split is much larger than the slit distance *a*, we get $C_{s1,2} \ll C_m$, then

$$C_1 \approx C_2 \approx \frac{C_m}{2} \tag{S7}$$

Since C_1 and C_2 are connected in series, the total capacitance of DSRR can be obtained by

$$C_s = \frac{C_1 C_2}{C_1 + C_2} = \frac{C_m}{4} = \frac{k_1 + k_2}{8\pi} \varepsilon_0 \varepsilon_{eff} \cosh^{-1}\left(\frac{2c}{d}\right) \tag{S8}$$

where ε_{eff} represents the effective permittivity determined by the substrate and chemical sample. By substituting the corresponding parameter values into Eq. (S8), the capacitance of resonator A and resonator B can be obtained as C_{SA}= 2.510 pF, C_{SB}= 3.232pF. The final values of resistance, inductance and capacitance are listed in Table S1.

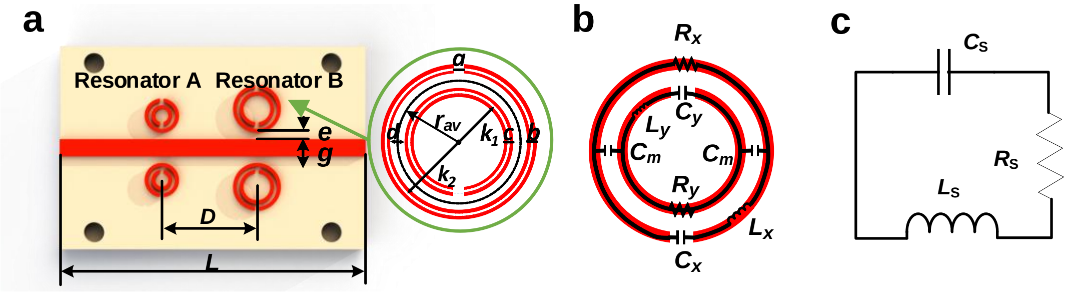

Figure S2. (a) A top view of the sensor with dimensions as follows: Resonator A: a_2=0.18, b_2=0.18, c_2=0.18, d_2=0.18, e_2=0.2, r_{av2}=0.8, unit: mm; Resonator B: a_1=0.2, b_1=0.2, c_1=0.2, d_1=0.2, e_1=0.2, r_{av1}=1.03, unit: mm; g=0.76, D=6.12, L=24, unit: mm. (b) Electrical components of DSRR as follows: resistance ($R_{x,y}$), capacitance ($C_{x,y,}$) and inductance ($L_{x,y}$) of the ring, the total resistance (*Rs*), capacitance (*Cs*), and inductance (*L*) of the DSRR. (c) Equivalent circuit of DSRR.

Table S1 The Analysis values of resistance, inductance and capacitance of equivalent circuit

Resonator	Inductance (pH)	Capacitance (pF)
Resonator A	151	2.510
Resonator B	165	3.232

3. Analysis of the relationship between dielectric properties and resonant frequency shifts.

According to Equation (1) in the manuscript, the resonance frequency shift is dependent on the capacitance and inductance of the device. The permeability of non-ferromagnetic materials such as alcohols and water is approximately equal to the vacuum permeability μ_0. Therefore, the resonance frequency shift depends only on the capacitance of the device. From Equation (S8), it is clear that since it is difficult to change the geometrical dimensions after fabrication, the capacitance is dependent on the effective permittivity around the resonator, which can be expressed as

$$\Delta C_{\varepsilon} = \frac{k_1 + k_2}{8\pi} \varepsilon_0 \Delta\varepsilon_{eff} \cosh^{-1}\left(\frac{2c}{d}\right) \tag{S9}$$

where ΔC_{ε} and $\Delta\varepsilon_{eff}$ represent the amount of change in capacitance and effective permittivity caused by the sample.

In order to investigate the relationship between dielectric properties and resonant frequency shifts, different $\Delta\varepsilon_{eff}$ are substituted into Equation (S9), and then the resonant frequency shift of resonator A is obtained by the equivalent circuit model in Figure 2c (in the manuscript), as shown in Figure S3. The reference frequency was set to the frequency when the microfluidic channel is empty ($\Delta\varepsilon_{effA} = 0$). Obviously, the resonant frequency f_0 decreases linearly as the permittivity $\Delta\varepsilon_{effA}$ increases, and the larger the change in the permittivity of the sample, the larger the resonant frequency shift. This is because the sample changes the effective permittivity around the resonator, which changes the capacitance of the device, causing resonant frequency shift. Therefore, by reducing the thickness of the substrate between the resonator and the microfluidic channel to enhance sensitivity, a larger resonant frequency shift can be obtained for

the same sample. Moreover, it is quite simple to estimate the resonance frequency shift of a sample by the permittivity. For example, the resonant frequency shift of water is expected to be greater than that of methanol and ethanol.

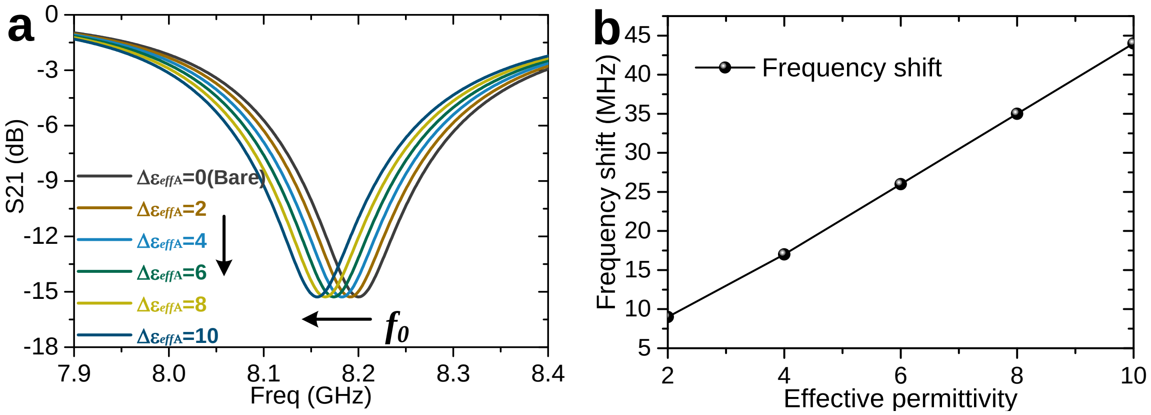

Figure S3. (a) Simulated S_{21} value of samples with different effective permittivity in channel A by circuit simulation software Advanced Design System. (b) The relationship between permittivity and resonant frequency shifts.

4. Sensitivity analysis of the MIM sensor

In the manuscript, the sensitivity S of the MIM sensor is defined as the slope of the concentration-frequency curve, which can be expressed as

$$S = \frac{\Delta f}{\Delta c} \propto \frac{\Delta f}{\Delta \varepsilon} \tag{S10}$$

where Δc , $\Delta \varepsilon$ and Δf represent the relative change in sample concentration, resonant frequency and permittivity, respectively. It means that the sensitivity can be enhanced by increasing the resonant frequency shift. The sensitivity of the MIM sensor can be analyzed by three factors: the geometry dimensions of the resonator, the shape of microfluidic channel, and the permittivity of the substrate. Since the design of the microfluidic channel has been discussed in the previous section, it will not be repeated here.

For the dimensions of the resonator, its impact on resonance is achieved by determining the electrical parameters of the resonator. From Equation (S2) and (S8), it is clear that the capacitance C_s and inductance L_s of the resonator are positively related to the radius of the resonator (k_1, k_2). Meanwhile, according to Equation (1) in the manuscript, the resonant frequency f_0 is inversely proportional to C_s and L_s. Therefore, it can be inferred that the larger the radius of the resonator, the smaller the resonance frequency. However, the sensitivity depends on the frequency shift rather than the resonant frequency, and the sensitivity of resonators A (2.0 MHz/1%) and B (2.1 MHz/1%) in the manuscript is almost the same. Therefore, there is no direct correlation between sensitivity and the dimensions of the resonator.

For the permittivity of the substrate, Rogers RO4003c ($\varepsilon_r = 3.38$), Rogers RO3006 ($\varepsilon_r = 6.15$) and Rogers RO3010 ($\varepsilon_r = 10.2$) were used as substrates and then simulated by finite

element simulation software. It is assumed that the channel is filled with ethanol (ε_r= 25), and the reference frequency was set to the frequency when the microfluidic channel is empty. Figure S4 shows that the smaller the permittivity of the substrate, the larger the resonance frequency shift. This is because the effective permittivity of substrate composed of low dielectric material is relatively easily to change as the sample enters the microfluidic channel. Therefore, it is feasible to use the material with a lower permittivity as a substrate to enhance the sensitivity of the sensor.

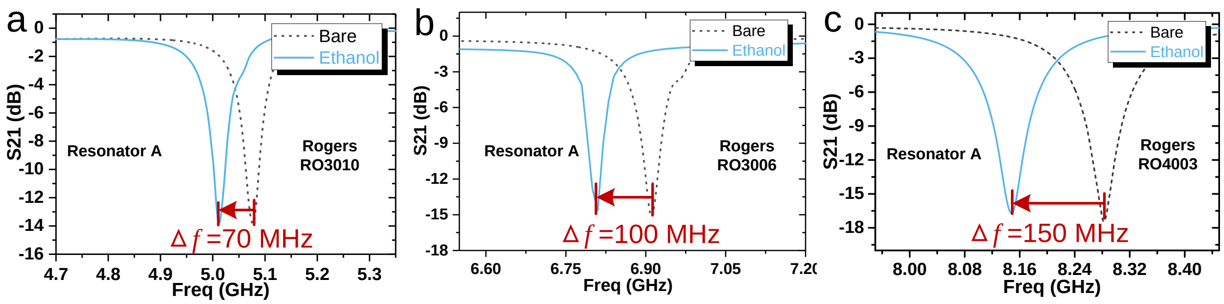

Figure S4. The simulated results of the sensor when the substrate is (a) Rogers RO3010, (b) Rogers RO3006 and (c) Rogers RO4003c.

5. Sensing test of alcohol-alcohol solution

The resonance occurring in the DSRR is dominated by the complex permittivity of the sample in the channel. When the permittivity of the binary mixture shows a good linear relation with the volume fraction of one of the components, the sensor can achieve a linear measurement, which indicates that it is also feasible to use alcohol-alcohol mixture like ethanol-methanol as the analyte. To verify this, the S21 spectrum of the sensor is simulated by finite element simulation software when the analyte is ethanol-methanol solution. As shown in Figure S5, the resonant frequency shifts linearly (R^2=0.995) as the volume fraction of methanol changes from 0% to 100% of 20% increment per step, indicating that alcohol-alcohol solution can also be measured by the sensor. It is worth noting that the permittivity of ethanol-methanol solution used in the simulation is from reference 3, as listed in Table S2.

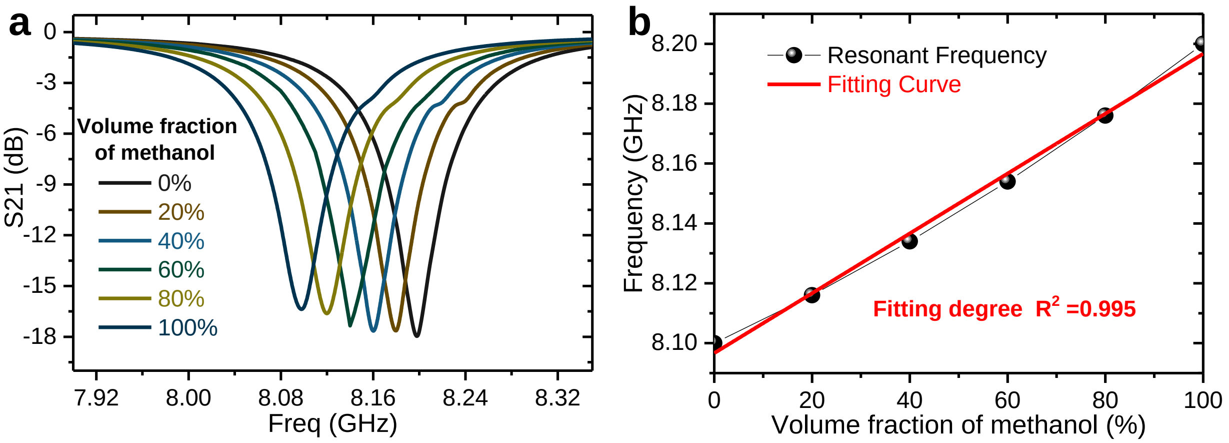

Figure S5. (a) Simulated S_{21} value of the sensor filled with ethanol-methanol solution in channel A, when the volume fraction of methanol changes from 0% to 100%. (b) Corresponding resonant frequency and fitting curve at different volume fraction of methanol in channel A.

Table S2 The permittivity of ethanol-methanol at 22 ℃ from reference [3]

Permittivity \ Volume fraction[a]	0%	20%	40%	60%	80%	100%
ε'	5.0	5.5	6.2	7.0	8.0	9.1
ε''	2.6	3.4	4.4	5.5	6.9	8.2

[a] Volume fraction of methanol.

6. Fabrication process of the MIM sensor

The detailed fabrication process is shown in the Figure S6. a) A 0.45 μm copper (Cu) film was deposited on a Rogers 4003c substrate using a magnetron sputtering system (JGP-450, SKY Technology Development Co., Ltd, China). b) A thin layer of positive photoresist (AZ 1500, AZ Electronics Materials) was spin coated on the surface of the substrate using KW-4A spinning coater (Institute of Microelectronics of Chinese Academy of Science, China). c) The photoresist was exposed to ultraviolet light through a mask with specific patterns. d) After the entire substrate was placed inside a developing solution (AZ400K, AZ Electronics Materials), the loosely bound photoactive compound layer irradiated with UV light was dissolved. e) The copper without photoresist protection was etched after the substrate had been put into the Cu etchant (ferric chloride, Meryer (Shanghai) Chemical Technology Co., Ltd.,). f) The remaining photoresist was removed in acetone. g) A thick layer of positive photoresist (AZ P4620, AZ Electronics Materials) was spin coated on the membrane surface. h) The photoresist was exposed to ultraviolet light through a mask with specific patterns. i) The entire substrate was placed inside a developing solution (AZ400K, AZ Electronics Materials). j) A 3 μm thick Ni layer was deposited on the copper pattern using the magnetron sputtering system. k) A 0.05 μm thick Au layer was deposited on the Ni pattern. l) The remaining photoresist was removed in acetone. a′~c′) Cu (0.45 μm), Ni (3 μm) and Au (0.05 μm) were successively deposited on the bottom surface of another Rogers 4003c substrate using the magnetron sputtering system(JGP-450, SKY Technology Development Co., Ltd, China). d′) The microfluidic channels were carved on the top surface of the substrate by craft-cutting technology. m) The components in l) and d′) were adhered together by epoxy adhesive to complete the fabrication of the sensor.

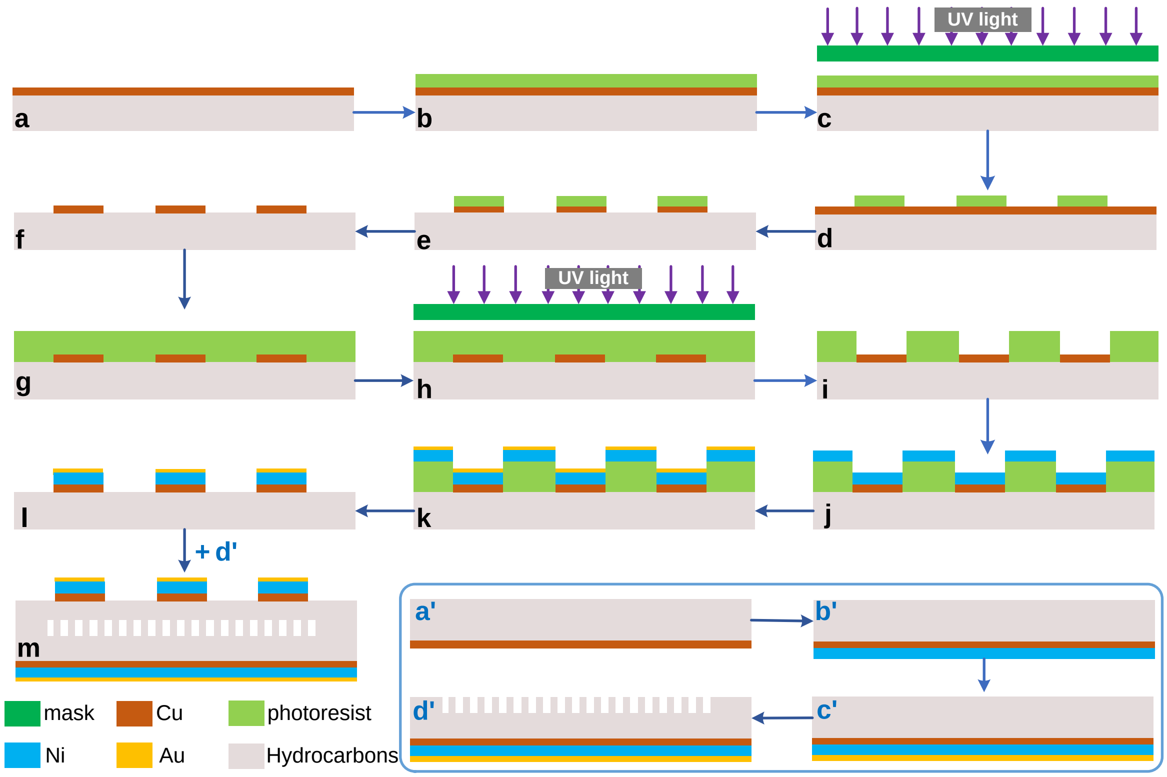

Figure S6. Fabrication processes of the MIM sensor.

7. The flow diagram for dual-band sensing of chemical

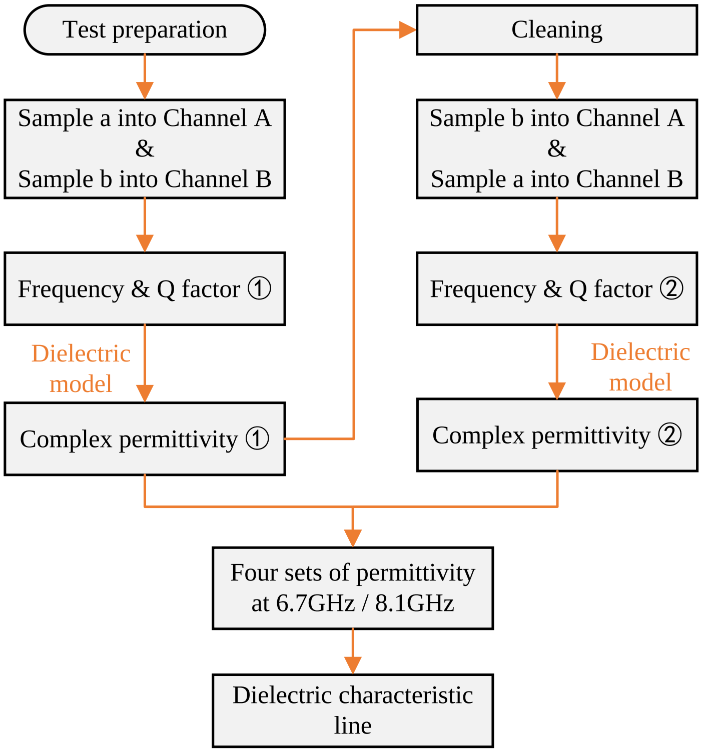

Figure S7. Dual-band sensing flow diagram for dielectric properties of chemicals.

8. The interference between resonators

Since the electromagnetic energy of the resonator is almost distributed at the split of the resonator, the interference induced by the adjacencies between the resonators can be considered to be small. To verify this hypothesis, the occurrence of resonance on the resonators is simulated by finite element simulation software, when the minimum spacing between resonators changes. Figure S8 shows the electric field distribution and the corresponding frequency response of resonators, when the minimum spacing between them changes from 0·λ to λ/4. The value of the wavelength is set to the larger resonant wavelength in the two resonators. The results show that only when the resonators are in electrical contact, there will be electrical distribution on both resonators at the same time, indicating that the interference between the two resonators was small before they were electrically contacted. Moreover, although the minimum spacing between resonators is different, their resonant frequency response is almost the same.

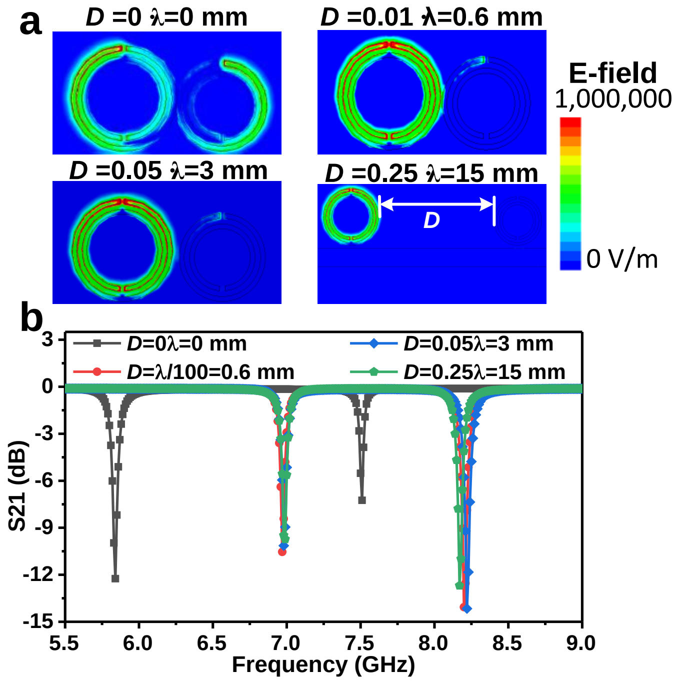

Figure S8. (a) The electric field distribution and (b) the corresponding frequency response of resonators, when the minimum spacing between them changes.

9. The dimension of each resonator of the multi-band sensor

Table S3 The dimension of each resonator of the multi-band sensor

Parameter[a]	Resonator 1 (mm)	Resonator 2 (mm)	Resonator 3 (mm)	Resonator 4 (mm)	Resonator5 (mm)
a	0.23	0.21	0.19	0.17	0.15
b	0.23	0.21	0.19	0.17	0.15
c	0.23	0.21	0.19	0.17	0.15
d	0.23	0.21	0.19	0.17	0.15
r_{av}	0.70	0.74	0.78	0.82	0.86

[a] Each parameter is labeled in Figure S2a.

Reference

[1] Hayes G. J. *et al.* Flexible Liquid Metal Alloy (EGaIn) Microstrip Patch Antenna. *IEEE Trans. Antennas Propag.* **60**. 2151-2156(2012).

[2] Sauviac B., Simovski C. R. & Tretyakov S. A., Double Split-Ring Resonators: Analytical Modeling and Numerical Simulations, Electromagnetics. 24. 317-38(2004).

[3] Bao, J. Z., Swicord, M. L. & Davis, C. C. Microwave dielectric characterization of binary mixtures of water, methanol, and ethanol. *J. Chem. Phys.* **104**, 4441-4450 (1996).
